# Multiomics-Based Outcome Prediction in Personalized Ultra-Fractionated Stereotactic Adaptive Radiotherapy (PULSAR)

**DOI:** 10.3390/cancers16193425

**Published:** 2024-10-09

**Authors:** Haozhao Zhang, Michael Dohopolski, Strahinja Stojadinovic, Luiza Giuliani Schmitt, Soummitra Anand, Heejung Kim, Arnold Pompos, Andrew Godley, Steve Jiang, Tu Dan, Zabi Wardak, Robert Timmerman, Hao Peng

**Affiliations:** 1 Department of Radiation Oncology, The University of Texas Southwestern Medical Center, Dallas, TX 75390, USA; 2 Medical Artificial Intelligence and Automation Laboratory, The University of Texas Southwestern Medical Center, Dallas, TX 75390, USA

**Keywords:** PULSAR, brain metastases, multiomics, outcome prediction, decision-making

## Abstract

**Simple Summary:**

Each patient responds uniquely to treatment, which makes personalized ultra-fractionated stereotactic adaptive radiotherapy (PULSAR) a promising strategy that delivers high-dose radiation at extended intervals for tailored adaptation. Currently, treatment modifications mainly rely on physicians’ assessments of tumor size changes. Our study aims to develop a more objective multiomics-based approach for predicting treatment outcomes in PULSAR, including radiomics, dosiomics, and delta features. By leveraging multiomics analysis and machine learning, we intend to transition the adaptation and decision-making process from empirical judgments to a more data-informed strategy, allowing clinicians to swiftly respond to changes in tumor behavior and provide more personalized treatment for each patient.

**Abstract:**

**Objectives**: This retrospective study aims to develop a multiomics approach that integrates radiomics, dosiomics, and delta features to predict treatment responses in brain metastasis (BM) patients undergoing PULSAR. **Methods**: A retrospective study encompassing 39 BM patients with 69 lesions treated with PULSAR was undertaken. Radiomics, dosiomics, and delta features were extracted from both pre-treatment and intra-treatment MRI scans alongside dose distributions. Six individual models, alongside an ensemble feature selection (EFS) model, were evaluated. The classification task focused on distinguishing between two lesion groups based on whether they exhibited a volume reduction of more than 20% at follow-up. Performance metrics, including sensitivity, specificity, accuracy, precision, F1 score, and the area under the receiver operating characteristic (ROC) curve (AUC), were assessed. **Results**: The EFS model integrated the features from pre-treatment radiomics, pre-treatment dosiomics, intra-treatment radiomics, and delta radiomics. It outperformed six individual models, achieving an AUC of 0.979, accuracy of 0.917, and F1 score of 0.821. Among the top nine features of the EFS model, six features came from post-wavelet transformation and three from original images. **Conclusions**: The study demonstrated the feasibility of employing a data-driven multiomics approach to predict treatment outcomes in BM patients receiving PULSAR treatment. Integrating multiomics with intra-treatment decision support in PULSAR shows promise for optimizing patient management and reducing the risks of under- or over-treatment.

## 1. Introduction

Brain metastases (BMs) are the most common type of intracranial tumors, affecting 20 to 40% of patients with systemic cancer [1,2,3,4]. Surgical intervention and radiotherapy have become the primary treatment options, as many chemotherapeutic agents cannot effectively cross the blood–brain barrier [5]. While surgical resection provides immediate relief from mass effects, it is often inadequate for eliminating multifocal microscopic disease. Stereotactic radiosurgery (SRS) employs highly focused radiation; however, its efficacy decreases with increasing tumor size and carries a greater risk of neurotoxicity [4,5,6]. The field has progressively transitioned toward fractionated stereotactic radiotherapy (fSRT) and staged stereotactic radiosurgery (SSRS) for larger brain metastases with the aim of improving local tumor control while reducing the risk of adverse radiation effects [7,8,9,10]. Nonetheless, the determination of optimal fractionation and intervals remains an active area of research [4].

At our institution, we implemented a similar approach to SSRS called personalized ultra-fractionated stereotactic adaptive radiotherapy (PULSAR), in which high-dose radiation is delivered at two- to four-week intervals. These extended intervals (weeks or months) allow for the enhanced recovery of normal tissues while providing sufficient time for the tumor and tumor microenvironment (TME) to undergo significant changes [11,12]. This enables more effective adaptation to the evolving tumor characteristics. Additionally, there is growing interest in the potential synergistic effects between PULSAR and checkpoint blockade inhibitor immunotherapy [11].

**Figure 1:**
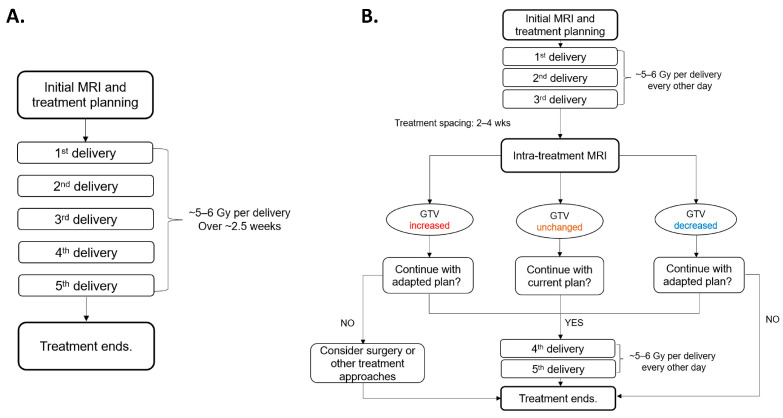
Comparison of workflows between (**A**) fractionated stereotactic radiotherapy (fSRT) and (**B**) PULSAR. PULSAR includes an intra-treatment MRI assessment to evaluate the change in GTV (increased, unchanged, or decreased), enabling more personalized treatment and timely adjustment.

To fully realize the potential of PULSAR, early decision-making is critical (Figure 1). The current process relies heavily on physician expertise, with treatment plans often adjusted based on gross tumor volume (GTV) changes observed in intra-treatment MRI scans [13,14,15]. However, this simple metric may not accurately reflect the overall treatment outcome. Therefore, developing a more objective and quantitative method to guide decision-making in PULSAR is essential [16,17]. To address this, our study employed a multiomics approach to predict treatment outcomes. In contrast to most previous studies that focus solely on pre-treatment imaging, our investigation integrated radiomics and dosiomics features extracted at multiple time points (i.e., a delta mode) to enhance predictive accuracy—an important aspect of PULSAR [18,19,20,21,22,23,24].

## 2. Materials and Methods

### 2.1. PULSAR Treatment, Study Population, and Data Acquisition

Figure 1 provides a workflow comparison between fSRT and PULSAR for patients with BMs undergoing radiosurgery utilizing Gamma Knife Icon™ (Elekta AB, Stockholm, Sweden). The patient initially undergoes a pre-treatment MRI scan, followed by the first treatment course comprising three fractions/pulses (5 to 6 Gy per fraction/pulse) with a two-day interval between fractions. After two to four weeks, the second course is delivered according to the intra-treatment MRI scans, with adjustments for changes in tumor volume and/or the presence of vasogenic edema. The plan may be adjusted to target a smaller GTV if it has shrunk or to accommodate an increasing target size through a dose boost or surgical intervention.

The retrospective study involved the examination of 39 BM patients who underwent PULSAR treatment at UTSW. The cohort comprised 69 lesions treated from 1 November 2021 to 1 May 2023, with both single and multiple BMs. Table 1 summarizes detailed demographic and clinical profiles, including age, gender, lesion number, and treatment specifics. Comprehensive initial and intra-treatment data were collected from patients undergoing PULSAR, encompassing MRI images, 3D dose maps (RTdose), and radiotherapy contour structure files (RTstructure). All collected images were acquired using axial (AX) MRI sequences with T1-weighted enhancement, ensuring a consistent and standardized basis for radiomic analysis. This approach minimized variations and potential discrepancies that could arise from different imaging modalities or sequences, thereby ensuring the accuracy of subsequent deltaomics calculations in the PULSAR cohort. Additionally, tumor volumes in follow-up MRI images were evaluated one to three months after PULSAR treatment. Treated BMs were manually contoured on these follow-up scans using the Research Velocity 4.1 platform (Varian Medical Systems, Inc., Palo Alto, CA, USA). A thorough comparison of pre-, intra-, and post-treatment images for each lesion was conducted by a research assistant and subsequently reviewed by an experienced physician. For all patients in this study, no new lesions were detected during the treatment intervals or at follow-up.

### 2.2. Data Processing and Multiomics Feature Extraction

Pre-processing steps involved an image resolution adjustment and co-registration (MRI image, RTstructure, RTdose). The 3D MRI images, 3D dose maps, and 3D GTVs were co-registered. Subsequently, they were re-sampled to standardize voxel sizes to a uniform 1 × 1 × 1 mm^3^, ensuring accuracy and consistency in feature extraction. Feature extraction was divided into the following two parts: (1) radiomics features: 1st radiomics features (from pre-treatment MRI) and 2nd radiomics features (from intra-treatment MRI) were extracted using PyRadiomics, with each scenario yielding 851 features and (2) dosiomics features: 3D dose maps were treated in the same manner as MRI images, with the extraction of 1st dosiomics features and 2nd dosiomics features using PyRadiomics, also yielding 851 features per scenario [25]. These features encompassed various categories, including intensity, shape, texture, and wavelet filters, providing a comprehensive evaluation of tumor and dose characteristics. Furthermore, delta radiomics and delta dosiomics features were computed by subtracting the first radiomics/dosiomics features from the second radiomics/dosiomics features after normalization.

### 2.3. Multi-Level Feature Selection

Given the high dimensionality of the radiomics and dosiomics feature sets, a multi-level feature selection strategy was implemented in three sequential steps. The first step was the filtering method, based on the Spearman’s correlation coefficients between each feature and the volume change. The top 50% of features with the highest absolute correlation values were retained for further analysis. The second step was the LASSO (least absolute shrinkage and selection operator) regression embedded method, which introduced an L1 regularization penalty term to the loss function for reducing the number of features [26]. The third step was recursive feature elimination combined with a support vector machine (SVM) classifier to rank features based on their importance and iteratively remove the least important ones [27,28]. To minimize potential bias and overfitting in feature selection, we conducted the above three steps with multiple train–test splits. For each split, feature selection was conducted solely on the training folds, and the top nine features were recorded. Finally, the nine most frequently occurring features were identified.

### 2.4. Predictive Model and Performance Evaluation

The model predicts if a lesion is going to show a volume reduction of ≥20% at follow-up. Several studies suggest a significant correlation between a reduction in volume of 20% or greater and the improvement of neurological signs and symptoms, reproducible when interpreted by different clinicians [13,14,15]. The same criterion was adopted to categorize lesions, framing it as a classification problem. Lesions with a follow-up tumor volume equal to or greater than 80% of their initial volume were classified as “non-decreased volume” (Group A), while those below the threshold were classified as “decreased volume” (Group B). We trained six individual models, comprising pre-treatment images (“1st radiomics”), intra-treatment images (“2nd radiomics”), the change between pre-treatment and intra-treatment radiomics (“delta radiomics”), pre-treatment dose distributions (“1st dosiomics”), intra-treatment dose distributions (“2nd dosiomics”), and the change between pre-treatment and intra-treatment dosiomics (“delta dosiomics”). Each model, utilizing the nine most frequent features identified in Section 2.3, was fitted using a support vector machine (SVM) for classification. Due to the small size of the dataset (as well as being imbalanced), each model was trained and validated via stratified 5-fold cross-validation, maintaining the ratio between the two groups as constant in each fold. To assess the robustness and stability of each model, the 5-fold cross-validation was iterated 50 times. Performance metrics, including sensitivity, specificity, accuracy, precision, F1 score, and AUC, were calculated for each iteration. Aggregated metrics across 50 iterations, such as mean values, standard deviations, and 95% confidence intervals, were used to evaluate the overall performance and performance fluctuations.

### 2.5. Ensemble Feature Selection (EFS)

To fully leverage the information available at the intra-treatment time point, which includes the pre-treatment image, intra-treatment image, and pre-treatment dose, an EFS strategy was implemented to combine the most critical features from four scenarios, namely 1st radiomics, 2nd radiomics, delta radiomics, and 1st dosiomics. Similar to the approach used for developing individual models, an ensemble model was developed, and the same quantitative analysis was conducted. To interpret the output of the SVM models probabilistically, Platt scaling was used to evaluate the prediction probability of each lesion [29].

## 3. Results

### 3.1. Treatment Response

**Figure 2:**
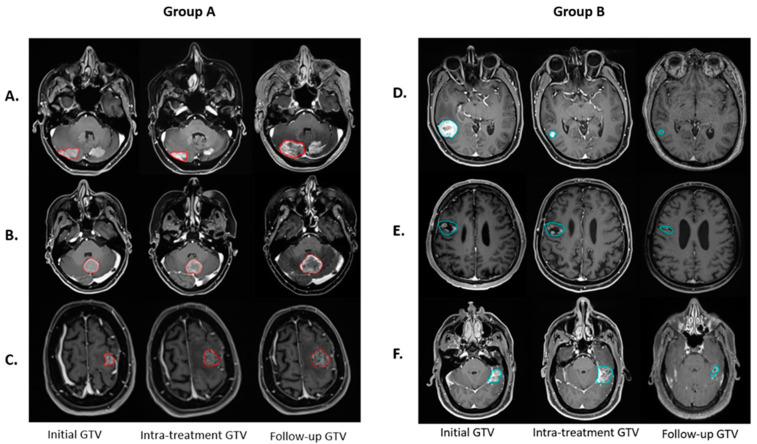
Six lesions illustrate the temporal evolution of GTV at various treatment stages. In Group A (lesions with red contours), three lesions exhibit non-decreased GTV at follow-up compared to the initial, but they display different GTV changes at the intra-treatment time point, with intra-treatment assessments of (**A**) decreased, (**B**) unchanged, and (**C**) increased GTV. In contrast, Group B (lesions with blue contours) depicts three lesions with a decreased GTV at follow-up compared to the initial, with intra-treatment variations categorized as (**D**) decreased, (**E**) unchanged, and (**F**) increased GTV.

Volumetric analysis demonstrates a complex response to PULSAR treatment among lesions. Figure 2 shows six representative lesions, each demonstrating the change in tumor volume at three time points. The dynamics suggest that intra-treatment evaluations alone do not adequately reveal the treatment outcome.

**Figure 3:**
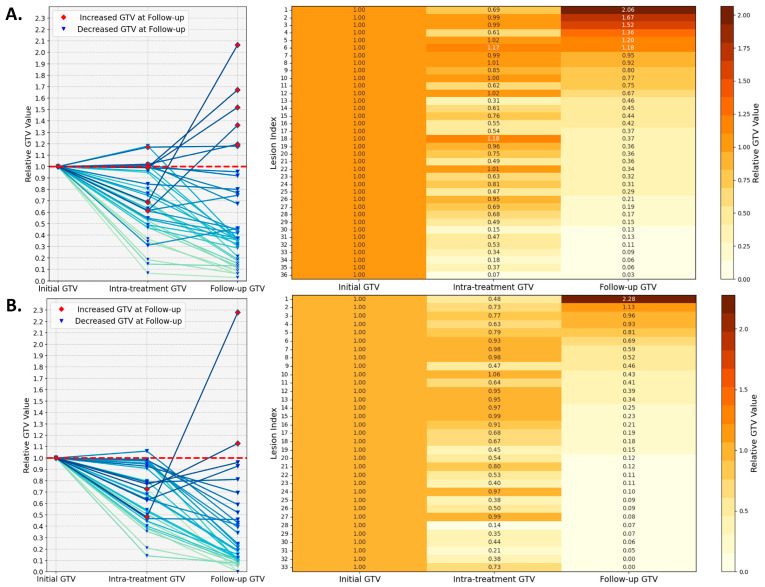
Lesion volumetric changes examined at three time points (pre-treatment, intra-treatment, and follow-up) for two subsets, namely lesions smaller than 4000 mm^3^ (**A**) and those larger than 4000 mm^3^ (**B**). The line graphs on the left display the relative GTV changes for each lesion during PULSAR. The accompanying heatmaps on the right provide a detailed quantitative representation of these changes, with color intensity reflecting the relative increase or decrease in tumor volume.

To quantify GTV change, Figure 3 maps out the volumetric dynamics of all 69 lesions at pre-treatment, intra-treatment, and follow-up time points, with the initial tumor volume normalized to one for each lesion. The analysis stratifies the lesions into the following two subsets based on a threshold of 4000 mm^3^: 36 large lesions (Figure 3A) and 33 small lesions (Figure 3B). Among the large lesions, five out of thirty-six exhibit an increase in intra-treatment volume, with the highest ratio of 1.18, and eight lesions display a ratio in a range between 0.8 and 1.0. The intra-treatment volume for the small lesions has a single case showing a ratio of 1.06, and the other 11 lesions are between 0.8 and 1.0. When examining the last column in the heatmaps (follow-up GTV), for the subset of large lesions, six lesions exhibit an increase, with the largest ratio of 2.06, while three lesions achieve a moderate reduction in volume, with the ratio between 0.8 and 1.0. In the small lesion subset, three lesions show minimal change, with ratios ranging from 0.8 to 1.0, while two lesions increase in size, with the largest ratio being 2.28.

### 3.2. Performances of Individual Models

**Figure 4:**
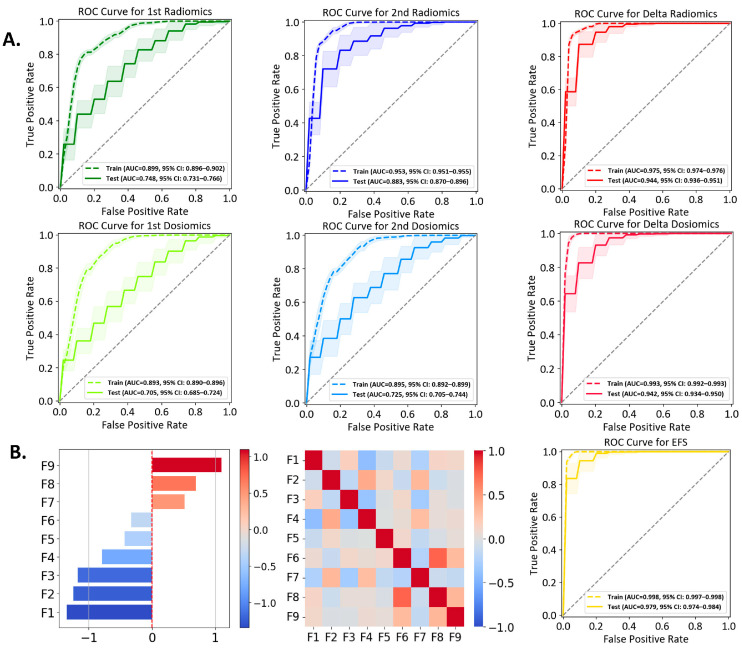
(**A**) ROC curves for six individual models. For each model, the plot shows the aggregated training (solid line) and test (dashed line) ROC curves generated from 50 iterations of 5-fold stratified cross-validation. The mean AUC with a 95% confidence interval is reported for each model. (**B**) Performance evaluation of the EFS model. The left panel displays the coefficient values of the nine selected features. The middle panel presents the correlation of the nine features. The right panel shows the ROC curve.

Fourteen lesions are classified into Group A and 55 lesions into Group B. Each model incorporates the top nine features after feature selection. The ROC curves for the six individual models are presented in Figure 4A and performance metrics are summarized in Table 2. Table 3 presents the pairwise *p*-values calculated using Welch’s *t*-test with Bonferroni correction for inter-model comparison, examining whether a significant difference exists between two models. Figure S1 shows the comparison between fivefold and threefold cross validation. Figure S2 illustrates the correlation among multiple features for each model, with the heatmaps providing an intuitive view of the relationships between the selected features.

In Figure 4A, the first dosiomics model exhibits the lowest AUC of 0.705, with a low precision of 0.285. The second dosiomics model exhibits the second-lowest AUC (0.725) and a low precision (0.319). Such inferior performance suggests the limitations of a model solely based on dosiomics. Two models based upon MRI image data, the first radiomics and the second radiomics models, outperform the above two counterparts, achieving AUC (0.748)/precision (0.344) and AUC (0.883)/precision (0.605), respectively. Compared to the first dosiomics model, the first radiomics model demonstrates improved performance, with significantly higher specificity, accuracy, AUC, and precision (*p*-values < 0.002, Table 3). The first radiomics and second dosiomics models demonstrate comparable performance, with no significant differences found between them. Among the four individual models based on single time points mentioned above, the second radiomics model yields the best performance.

The delta radiomics and delta dosiomics models show comparable performance, both outperforming the single time point models. The delta radiomics model excels in sensitivity (0.903 vs. 0.802), while the delta dosiomics model has higher specificity (0.836 vs. 0.872). Both models have a similar accuracy (0.850 vs. 0858), AUC (0.944 vs. 0.942), precision (0.632 vs. 0.661), and F1 score (0.720 vs. 0.694). The relative change in dose distribution provides valuable insights for classification. By leveraging temporal changes in radiomics features, the two delta models more effectively capture tumor evolution and treatment response.

### 3.3. Ensemble Feature Selection (EFS) Model

The EFS model comprises nine features identified from a pool of features originating from four scenarios (first radiomics, second radiomics, delta radiomics, and first dosiomics). The correlation heatmap of these nine features, shown in Figure 4B, demonstrates minimal inter-feature correlation. Detailed descriptions (e.g., names, types, weights in the SVM) are summarized in Table 4, where the weight coefficients (in a descending order) reflect their respective influence along with the z-score. The ROC curve of the EFS model is depicted in Figure 4B, exhibiting an AUC of 0.979, sensitivity (0.907), specificity (0.920), accuracy (0.917), precision (0.786), and F1 score (0.821). While the EFS model has a sensitivity comparable to that of the delta radiomics model, the EFS model achieves superior performance in all other metrics (Table 2). The ROC comparison of different models, as well as one example of the confusion matrices from a single iteration of the fivefold cross-validation, are provided in Figures S1 and S3, respectively.

### 3.4. Multiomics Feature Interpretation

**Figure 5:**
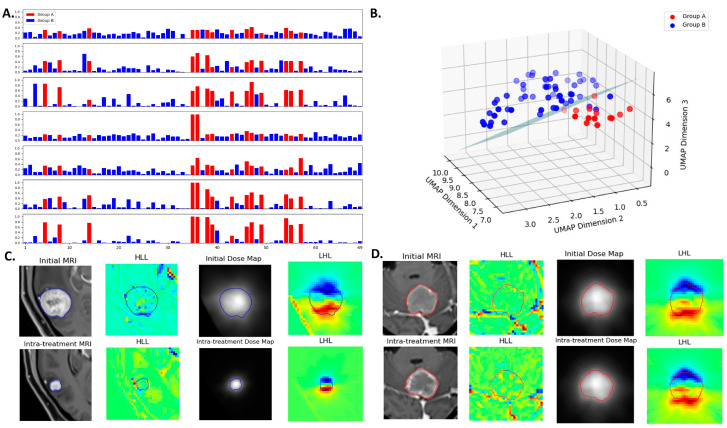
(**A**) Probability scores for lesions obtained across different models. The horizontal axis represents the lesion index, and the vertical axis represents the probability score of a lesion under different models. The models (from top to bottom) are 1st radiomics, 2nd radiomics, delta radiomics, 1st dosiomics, 2nd dosiomics, delta dosiomics, and the EFS model. The EFS model shows the most effective separation between the two groups. (**B**) UMAP visualization of the EFS model’s nine features, projected into a three-dimensional space to illustrate the separation between Group A and Group B lesions. An SVM hyperplane serves as the decision boundary between the groups. (**C**) A lesion (contour in blue) with decreased GTV at follow-up compared to the initial. (**D**) A lesion (contour in red) with non-decreased GTV at follow-up compared to the initial. Panels include initial MRI, intra-treatment MRI, initial dose map, intra-treatment dose map, and wavelet-transformed images.

The probability scores obtained using the method of Platt scaling are presented in Figure 5A, which demonstrates the varying degrees of discrimination among models. Higher values for Group A and lower values for Group B indicate better separability between the two classes. The mean probability scores for Group A versus Group B are as follows (from top to bottom in Figure 5A): 0.322 ± 0.070 vs. 0.187 ± 0.090 for the first radiomics model, 0.510 ± 0.167 vs. 0.145 ± 0.132 for the second radiomics model, 0.614 ± 0.167 vs. 0.129 ± 0.098 for the delta radiomics model, 0.350 ± 0.255 vs. 0.176 ± 0.056 for the first dosiomics model, 0.356 ± 0.132 vs. 0.170 ± 0.101 for the second dosiomics model, 0.565 ± 0.204 vs. 0.103 ± 0.093 for the delta dosiomics model, and 0.720 ± 0.196 vs. 0.073 ± 0.053 for the EFS model. Figure 5B visualizes the feature space using UMAP dimensionality reduction. The nine features from the EFS model are visualized using UMAP projection in a three-dimensional space to illustrate the separation between the two groups, with an SVM hyperplane serving as the decision boundary between the two groups.

Figure 5C,D display results for two lesions, featuring MRI and dose maps alongside wavelet-transformed images to aid in understanding the multiomics feature extraction. Table 4 outlines the nine features of the EFS model, primarily derived from radiomics features post-wavelet transformation (only three features directly from the original images). The 3D discrete wavelet transform decomposes volumetric images into multi-resolution components, capturing both fine and coarse details, with “L” representing low-pass and “H” representing high-pass filtering [30]. Features F2 and F9, with the highest weight coefficients within the SVM model (F2: −1.24, F9: 1.10), are elaborated on below.

Feature F2, known as low gray-level zone emphasis (LGLZE), quantifies the prevalence of low gray-level size zones and reflects the spatial uniformity in dose maps. The LGLZE value is 0.35 for a decreased GTV (Figure 5C) and 0.61 for a non-decreased GTV (Figure 5D). A lower LGLZE value suggests fewer low-dose regions within the tumor, potentially correlating with a favorable treatment response. Feature F9, derived from Kurtosis calculated in the delta mode based on radiomics, provides insights into evolving tumor heterogeneity during treatment. The lesions in Figure 5C,D exhibit delta Kurtosis values of 0.21 and 0.36, respectively. A smaller delta Kurtosis value indicates a reduction in lesion volume after treatment, whereas a larger delta Kurtosis value suggests an increase in volume.

## 4. Discussion

This preliminary study investigates the use of multiomics to predict treatment outcomes and support decision-making in PULSAR, using BMs as a case study. By integrating delta multiomics with SVM classification, we aim to improve predictive accuracy. As expected, the EFS model, which combines features from pre-treatment radiomics, pre-treatment dosiomics, intra-treatment radiomics, and delta radiomics, outperforms six individual models. In addition to PULSAR, our proposed framework can be applied to other forms of fSRT and SSRS.

The perspective on extending treatment time intervals continues to be a subject of debate, given the complex challenge of balancing adverse effects with effective tumor control. Both PULSAR and SSRS require careful personalization, given the significant variability in individual patient responses to treatment. While some patients may experience tumor reduction, others may demonstrate tumor growth. In addition, it is crucial to distinguish between pseudo-progression and true progression to guide subsequent treatment decisions. Additional factors, such as tumor radioresistance and characteristics associated with systemic therapy, further complicate treatment options. These variations highlight the necessity of closely monitoring each patient’s progress and adjusting treatment strategies as required. By employing a multiomics analysis and machine learning, we aim to transition the decision-making process from empirical judgments to a more data-driven approach, ensuring that each patient receives tailored treatment.

A notable characteristic of PULSAR is the availability of intra-treatment MRI images after a prolonged interval, which allows for the effective utilization of deltaomics. This capability is critical in the development of the EFS model, which demonstrates enhanced performance compared to traditional radiomics models that rely exclusively on pre-treatment data. This observation is consistent with prior research findings [22,23,24]. In constructing the EFS model, we assessed the contributions of various feature sets but chose to exclude features linked to the second dosiomics and delta dosiomics models for two pragmatic reasons. First, from a decision-making perspective, delta dosiomics and second dosiomics data would not be accessible prior to a physician’s new dose prescription and treatment plan formulation. Second, the performance of delta dosiomics is largely comparable to that of delta radiomics, suggesting that the added advantages of incorporating delta dosiomics may be minimal. This analysis of different models raises the following two relevant questions: (1) how does the integration of delta radiomics features improve performance? (2) What additional insights can dosiomics provide?

For the first question, we believe that delta radiomics features effectively capture the evolution of anatomical characteristics within GTV. The inclusion of temporal changes enhances predictive accuracy, as evidenced by the improved performance of both delta radiomics and second radiomics in comparison to first radiomics. In simpler terms, assessing treatment response is most effective when conducted after the treatment has been administered. Although Gao et al. [24] did not monitor dynamic changes during treatment as our PULSAR study did, they investigated radiomics features derived from longitudinal diffusion-weighted imaging (DWI) to predict post-treatment tumor necrosis. Their findings indicated that delta features calculated from pre- and post-treatment scans were more predictive than static features. Similarly, Wang et al. [23] explored the prognostic significance of delta radiomics features in predicting treatment response and clinical outcomes, including overall survival and disease-free survival. Their study also demonstrated that delta radiomics features outperformed single-time-point radiomics models.

For the second question, dosiomics features derived from 3D dose distributions may offer advantages over traditional dose–volume histogram (DVH) analyses [20,31]. By providing a detailed pixel-wise representation of the radiation dose, dosiomics effectively captures patient-specific dose heterogeneity within the treatment target [32]. Our findings indicate that delta dosiomics surpasses both second and first dosiomics, suggesting that changes in pre- and post-treatment dose maps—likely in low dose regions and dose fall-off areas—yield valuable insights. However, it is important to note that features from delta radiomics and delta dosiomics may overlap, allowing either feature set to be utilized for classification with comparable accuracy.

In this study, we focused extensively on feature selection and minimizing redundancy. To facilitate a fair comparison among different models while adhering to the guideline of maintaining a feature-to-observer ratio of at most 4:1, we selected the top nine features for each model [33]. The initial raw feature set consisted of 851 features, which posed a considerable challenge, as outlined in Section 2.3. Traditional feature selection techniques commonly utilize a two-sample *t*-test to identify features with the lowest *p*-values or employ multi-tiered approaches like Lasso regression and Welch’s *t*-test [34,35]. Our analysis, detailed in Table 4, reveals that only six of the nine selected features have significant *p*-values below 0.05 when assessed using the Mann–Whitney U test. The remaining three features, though individually not statistically significant, have positive SVM coefficients (F7, F8, and F9). This indicates that these features, despite their high *p*-values—attributable to either a small sample size or other factors—continue to make meaningful contributions to the model’s classification performance when combined with other features. Additional clarification on how fivefold cross-validation and frequency-based feature selection collectively mitigate overfitting and bias is provided in Figure S4.

In the EFS model, the top nine features were identified, as detailed in Table 4. While features such as tumor size or shape (F1) are straightforward, others derived from texture matrices and wavelet filtering present more complex interpretations. These features may be associated with tissue heterogeneity or dosiomics gradients, with the wavelet transform enabling multi-resolution decomposition and noise reduction [30,36]. Evaluating their sensitivity to variations in imaging protocols across different institutions needs to be another important area of research. The next phase of our study will focus on enhancing interpretability by establishing correlations between these features and other pathological and clinical characteristics. Notably, integrating delta changes in biomarkers that indicate responses to immunotherapy into the prediction model may leverage synergistic effects, thereby capitalizing on another unique benefit of PULSAR treatment [11,37,38]. Furthermore, while the current focus is on features extracted from within the GTV, significant insights may also emerge from the peritumoral region. For instance, gradient maps in the lesion’s periphery could serve as promising indicators of treatment response [39,40]. Additionally, exploring CNN-based autoencoders holds potential for feature extraction and may yield more interpretable results than traditional multiomics approaches.

Our study has several limitations that warrant further investigation. First, the small and retrospective patient cohort, a consequence of the early phase of the PULSAR trial, resulted in a limited number of cases that met the stringent recruitment criteria. The imbalanced dataset and lack of a fully independent validation dataset pose additional challenges. Efforts should be directed toward increasing the cohort size and enhancing the robustness of the predictions. Second, tumor size was chosen as the predictive outcome, with a 20% threshold established for classifying two groups based on the existing literature [13,14,15,41]. Further research is needed to assess how changes in tumor volume correlate with local control and other important clinical outcomes in the treatment of brain metastases. Additional clinical evidence is also required to validate the significance of a 20% change as a meaningful threshold; alternative thresholds (e.g., 30%, 50%) may be explored as well. Additionally, tumor necrosis may complicate tumor volume measurements. A tumor may appear to shrink due to necrosis, which does not necessarily indicate the complete eradication of viable cancer cells. Finally, the focus should not be solely on tumor volume change. Long-term outcomes such as overall survival, disease progression, and quality of life after treatment should also be considered.

## 5. Conclusions

Not all cancers are the same, and each patient responds differently to treatment. Using brain metastases as a case study, we developed a multiomics approach that integrates radiomics, dosiomics, and delta features for outcome prediction. The EFS model exhibits superior performance compared to six individual models, highlighting the significance of incorporating both pre-treatment and intra-treatment data in a delta mode. Our proposed framework can serve as a valuable tool for both PULSAR and other forms of fSRT/SSRS. Its potential clinical benefits include facilitating more timely and personalized treatment plan updates and reducing the risks associated with under- or over-treatment.

## Figures and Tables

**Table 1:** Patient demographics and lesion characteristics in PULSAR treatment. * Threshold for change, defined as a 20% GTV reduction.

Characteristics	Value
**Patient Information**	
Age (range)	61 (28–84)
Total Patients (Male: Female)	39 (14:25)
**Lesion Information**	
Single brain metastases	26
Multiple brain metastases	43
Total Lesions (Decreased: Non-Decreased) *	69 (55:14)
**Lesion size**	
Initial Tumor Volume (mm^3^)	3666.3 (14.5–37607.6)
3D Diameter (mm)	25.5 (3.7–79.8)
**Delivered Dose and Fractionation by Lesion Count (Gy, Fx)**	
30 Gy, 5 Fx	57
27.5 Gy, 5 Fx	2
25 Gy, 5 Fx	9
24 Gy, 4 Fx	1

**Table 2:** Performance metrics of different models.

	1st Radiomics	2nd Radiomics	Delta Radiomics	1st Dosiomics	2nd Dosiomics	Delta Dosiomics	Ensemble Feature Selection (EFS)
Sensitivity	0.503 ± 0.271 (95% CI: 0.469–0.537)	0.755 ± 0.266 (95% CI: 0.722–0.789)	0.903 ± 0.174 (95% CI: 0.882–0.925)	0.559 ± 0.271 (95% CI: 0.525–0.592)	0.538 ± 0.309 (95% CI: 0.499–0.577)	0.802 ± 0.242 (95% CI: 0.772–0.832)	0.907 ± 0.169 (95% CI: 0.886–0.928)
Specificity	0.745 ± 0.128 (95% CI: 0.729–0.761)	0.854 ± 0.102 (95% CI: 0.841–0.867)	0.836 ± 0.117 (95% CI: 0.822–0.851)	0.636 ± 0.141 (95% CI: 0.618–0.654)	0.713 ± 0.129 (95% CI: 0.697–0.729)	0.872 ± 0.102 (95% CI: 0.860–0.885)	0.920 ± 0.085 (95% CI: 0.909–0.931)
Accuracy	0.696 ± 0.107 (95% CI: 0.682–0.709)	0.833 ± 0.090 (95% CI: 0.822–0.844)	0.850 ± 0.094 (95% CI: 0.838–0.862)	0.621 ± 0.112 (95% CI: 0.607–0.635)	0.677 ± 0.111 (95% CI: 0.664–0.691)	0.858 ± 0.086 (95% CI: 0.847–0.869)	0.917 ± 0.072 (95% CI: 0.908–0.926)
AUC	0.748 ± 0.143 (95% CI: 0.731–0.766)	0.883 ± 0.107 (95% CI: 0.870–0.896)	0.944 ± 0.061 (95% CI: 0.936–0.951)	0.705 ± 0.157 (95% CI: 0.685–0.724)	0.725 ± 0.158 (95% CI: 0.705–0.744)	0.942 ± 0.062 (95% CI: 0.934–0.950)	0.979 ± 0.039 (95% CI: 0.974–0.984)
Precision	0.344 ± 0.197 (95% CI: 0.319–0.368)	0.605 ± 0.232 (95% CI: 0.577–0.634)	0.632 ± 0.197 (95% CI: 0.607–0.657)	0.285 ± 0.140 (95% CI: 0.268–0.302)	0.319 ± 0.182 (95% CI: 0.296 –0.341)	0.661 ± 0.224 (95% CI: 0.633–0.689)	0.786 ± 0.201 (95% CI: 0.761–0.811)
F1 Score	0.389 ± 0.193 (95% CI: 0.365–0.413)	0.640 ± 0.199 (95% CI: 0.615–0.665)	0.720 ± 0.151 (95% CI: 0.701–0.739)	0.366 ± 0.162 (95% CI: 0.346–0.386)	0.386 ± 0.205 (95% CI: 0.361–0.412)	0.694 ± 0.186 (95% CI: 0.671–0.718)	0.821 ± 0.147 (95% CI: 0.803–0.839)

**Table 3:** Pairwise comparison of model performance metrics. * Significant difference identified based on Welch’s *t*-test with Bonferroni correction.

	1st Radiomics vs. 2nd Radiomics	1st Radiomics vs. Delta Radiomics	1st Radiomics vs. 1st Dosiomics	1st Radiomics vs. 2nd Dosiomics	1st Radiomics vs. Delta Dosiomics	1st Radiomics vs. EFS	2nd Radiomics vs. Delta Radiomics	2nd Radiomics vs. 1st Dosiomics	2nd Radiomics vs. 2nd Dosiomics	2nd Radiomics vs. Delta Dosiomics	2nd Radiomics vs. EFS	Delta Radiomics vs. 1st Dosiomics	Delta Radiomics vs. 2nd Dosiomics	Delta Radiomics vs. Delta Dosiomics	Delta Radiomics vs. EFS	1st Dosiomics vs. 2nd Dosiomics	1st Dosiomics vs. Delta Dosiomics	1st Dosiomics vs. EFS	2nd Dosiomics vs. Delta Dosiomics	2nd Dosiomics vs. EFS	Delta Dosiomics vs. EFS
Sensitivity	<0.002 *	<0.002 *	**0.022**	**0.176**	<0.002 *	<0.002 *	<0.002 *	<0.002 *	<0.002 *	**0.041**	<0.002 *	<0.002 *	<0.002 *	<0.002 *	**0.829**	**0.428**	<0.002 *	<0.002 *	<0.002 *	<0.002 *	<0.002 *
Specificity	<0.002 *	<0.002 *	<0.002 *	**0.006**	<0.002 *	<0.002 *	**0.070**	<0.002 *	<0.002 *	**0.047**	<0.002 *	<0.002 *	<0.002 *	<0.002 *	<0.002 *	<0.002 *	<0.002 *	<0.002 *	<0.002 *	<0.002 *	<0.002 *
Accuracy	<0.002 *	<0.002 *	<0.002 *	**0.063**	<0.002 *	<0.002 *	**0.045**	<0.002 *	<0.002 *	<0.002 *	<0.002 *	<0.002 *	<0.002 *	**0.328**	<0.002 *	<0.002 *	<0.002 *	<0.002 *	<0.002 *	<0.002 *	<0.002 *
AUC	<0.002 *	<0.002 *	<0.002 *	**0.079**	<0.002 *	<0.002 *	<0.002 *	<0.002 *	<0.002 *	<0.002 *	<0.002 *	<0.002 *	<0.002 *	**0.758**	<0.002 *	**0.158**	<0.002 *	<0.002 *	<0.002 *	<0.002 *	<0.002 *
Precision	<0.002 *	<0.002 *	<0.002 *	0.140	<0.002 *	<0.002 *	**0.170**	<0.002 *	<0.002 *	**0.007**	<0.002 *	<0.002 *	<0.002 *	**0.127**	<0.002 *	**0.022**	<0.002 *	<0.002 *	<0.002 *	<0.002 *	<0.002 *
F1 Score	<0.002 *	<0.002 *	**0.152**	**0.882**	<0.002 *	<0.002 *	<0.002 *	<0.002 *	<0.002 *	<0.002 *	<0.002 *	<0.002 *	<0.002 *	**0.088**	<0.002 *	**0.221**	<0.002 *	<0.002 *	<0.002 *	<0.002 *	<0.002 *

**Table 4:** Summary of the EFS features. * Significant difference identified based on the Mann–Whitney U test.

Abbreviation	Multi-Omics Type	Wavelet Filtering/Original	Feature Class	Feature Name	Feature Weight Coefficient	Feature Values (Z-Score)		
						Group A	Group B	*p*-Value
F1	Delta Radiomics	Original	shape	Least Axis Length	−1.34382	−0.231 ± 0.233	0.059 ± 0.468	<0.05 *
F2	1st Dosiomics	Wavelet-LHL	glszm	Low Gray Level Zone Emphasis	−1.23960	−0.58 ± 0.867	0.148 ± 0.994	<0.05 *
F3	2nd Radiomics	Wavelet-LHH	glcm	Correlation	−1.17479	−0.577 ± 0.584	0.147 ± 1.043	<0.05 *
F4	2nd Radiomics	Original	glrlm	Long Run Low Gray Level Emphasis	−0.78866	−0.444 ± 0.195	0.113 ± 1.098	<0.05 *
F5	Delta Radiomics	Wavelet-HHH	gldm	Large Dependence High Gray Level Emphasis	−0.32663	−0.597 ± 0.951	0.152 ± 0.765	<0.05 *
F6	Delta Radiomics	Wavelet-HHH	glszm	Gray Level NonUniformity	−0.43211	−0.379 ± 0.674	0.096 ± 0.497	<0.05 *
F7	1st Radiomics	original	glcm	MCC	0.51466	0.146 ± 0.812	−0.037 ± 1.054	0.486
F8	Delta Radiomics	Wavelet-LLH	firstorder	Median	0.69508	0.558 ± 1.214	−0.142 ± 1.086	0.064
F9	Delta Radiomics	Wavelet-HLL	firstorder	Kurtosis	1.09794	0.254 ± 0.831	−0.065 ± 0.326	0.181

## Data Availability

The data presented in this study are available upon request from the corresponding author.

## Supplementary Materials

The following supporting information can be downloaded at: https://www.mdpi.com/article/10.3390/cancers16193425/s1, Figure S1: Performance comparison between 5-fold cross-validation and 3-fold cross-validation. Figure S2: Correlation heatmaps showcasing the final nine features selected for each predictive model after a comprehensive multi-level feature selection process. Figure S3: Confusion matrices from a single iteration of 5-fold cross-validation for the ensemble feature selection (EFS) model. Figure S4: Illustration of stratified 5-fold cross-validation along with frequency-based feature selection for mitigating overfitting and bias.
