# Supplementary material for: Multiomics-Based Outcome Prediction in Personalized Ultra-Fractionated Stereotactic Adaptive Radiotherapy (PULSAR)
Source: Cancers (Basel). 2024 Oct 9;16(19):3425. doi: 10.3390/cancers16193425 (PMC11475788; doi:10.3390/cancers16193425)
Supplement: Supplementary file 1 [file cancers-16-03425-s001.zip › cancers-3218483-supplementary.pdf]

## Supplemental Materials:

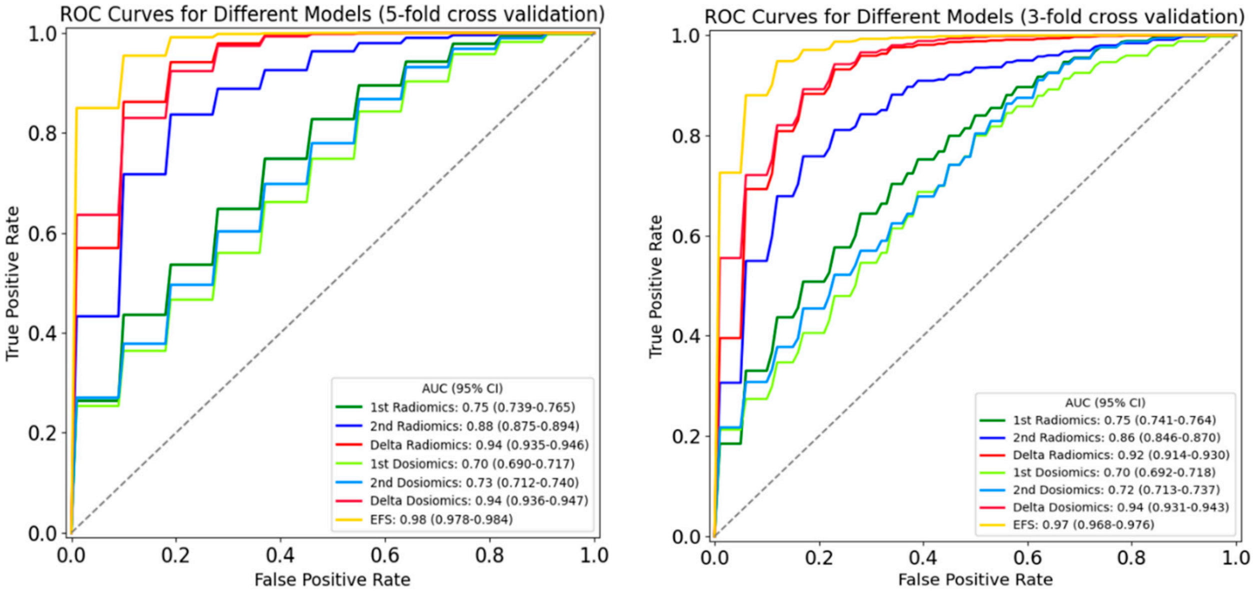


**Figure S1.** Performance comparison between 5-fold cross-validation and 3-fold cross-validation. Aggregated metrics from the five folds were used to evaluate overall performance fluctuation statistics, including mean values, standard deviations, and 95% confidence intervals. Each cross-validation was repeated 50 times with random seeds to assess the robustness and stability of each model.

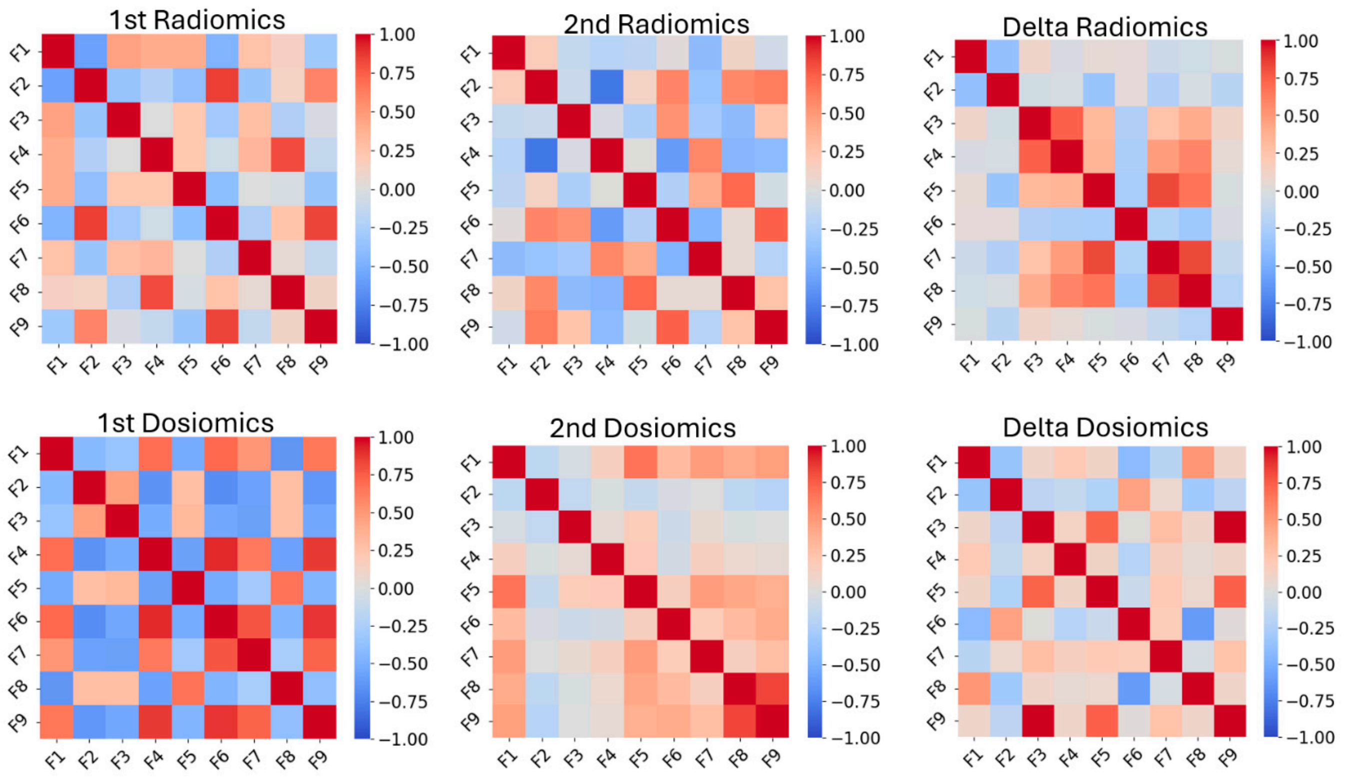


**Figure S2.** Correlation heatmaps showcasing the final nine features selected for each predictive model after the feature selection steps. The top row displays the heatmaps for the 1st radiomics, 2nd radiomics, and delta-radiomics models, while the bottom row corresponds to the 1st dosiomics, 2nd dosiomics, and delta-dosiomics models.

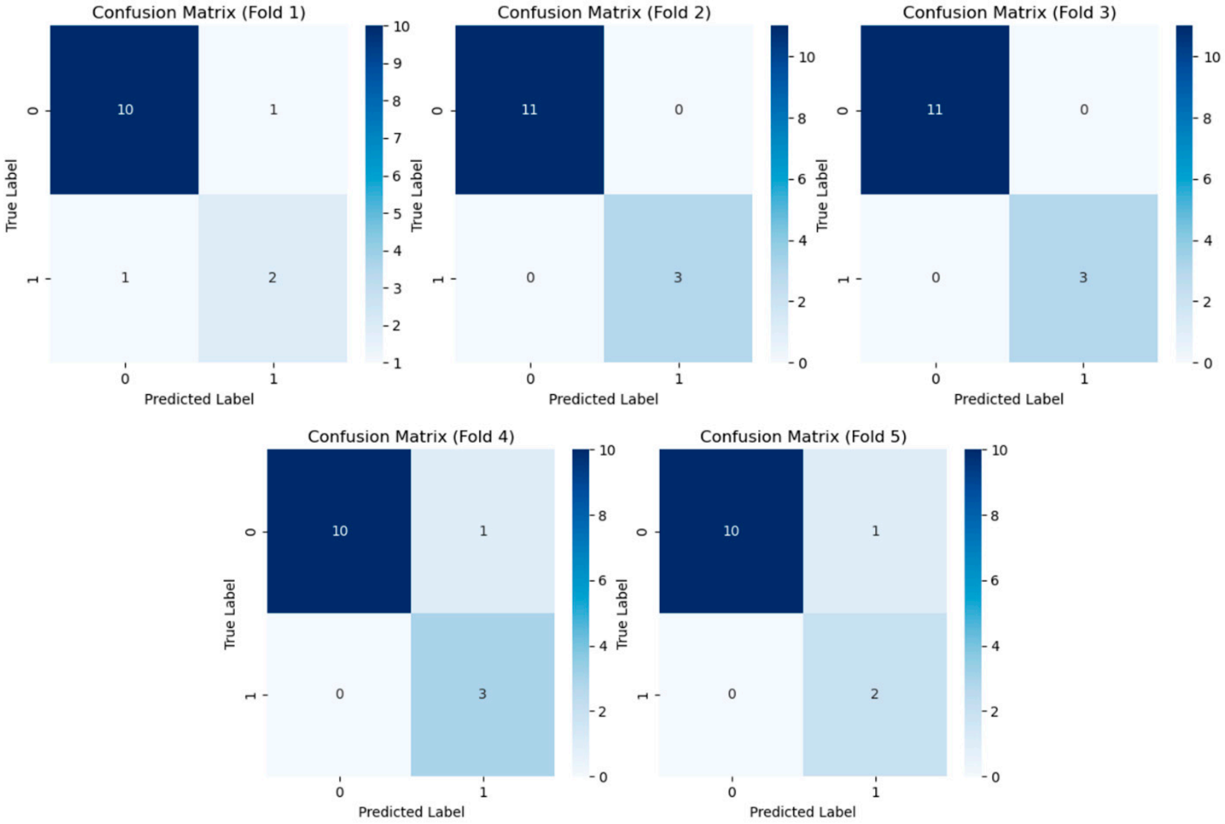


**Figure S3.** Confusion matrices from a single iteration of 5-fold cross-validation for the ensemble feature selection (EFS) model.

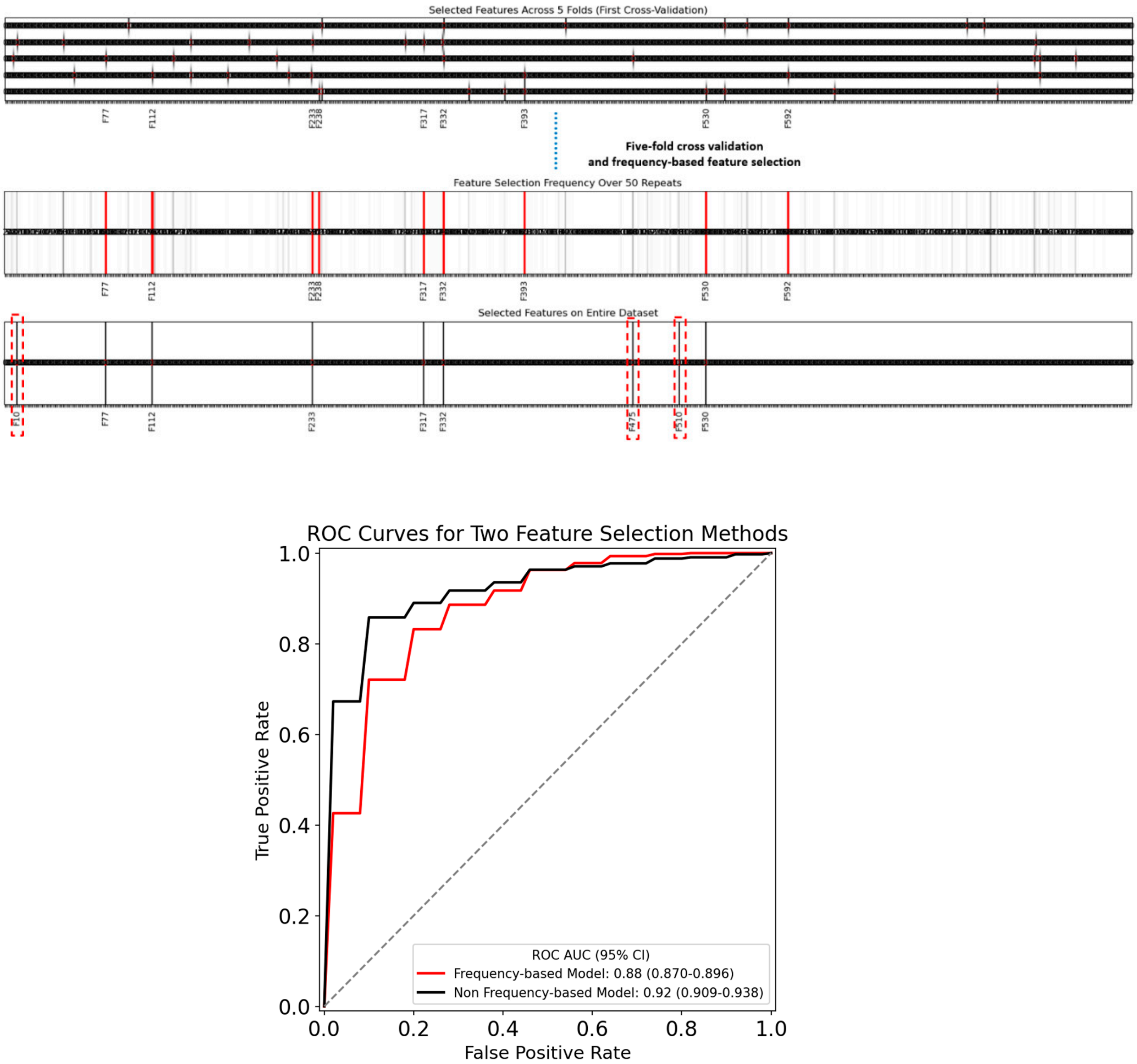


**Figure S4.** (Top): Illustration of 5-fold cross-validation (50 iterations) along with frequency-based feature selection to mitigate overfitting. Compared to the features selected based on the entire dataset (non-frequency-based model), only 6 out of 9 features overlap (excluding F10, F475, and F510). (Bottom): The AUC decreases from 0.92 (0.909–0.938) to 0.88 (0.870–0.896). We hypothesize that with additional data to form a complete independent testing dataset, the performance metrics will vary by no more than 5% from those reported in this preliminary study.

Ideally, having a larger dataset and an independent test set would be preferable. However, since PULSAR is new at our institute and data collection takes time, it is not currently possible for us to have a separate test set. Therefore, we have adopted frequency-based feature selection as a compromise to mitigate overfitting and information leakage. The benefits include: 1) **Avoiding Overfitting**: By performing multiple iterations of cross-validation and recording how frequently each feature is selected, we identify features that consistently contribute to the model's performance. This reduces the risk of overfitting to any specific subset of the data, as only robust features that generalize well across different folds are chosen. 2) **Reducing Variability**: Small datasets can lead to high variance in feature selection, where different features might appear important in different folds. Frequency-based selection mitigates this by focusing on features that repeatedly show their

importance, leading to a more reliable feature set. 3) **Minimizing Noise:** By selecting features that consistently contribute to the model's performance, we reduce the likelihood of including noisy or irrelevant features.
